# Neural therapy of an athlete’s chronic plantar fasciitis: a case report and review of the literature

**DOI:** 10.1186/s13256-018-1770-4

**Published:** 2018-08-21

**Authors:** J. Fleckenstein, M. König, W. Banzer

**Affiliations:** 1 Department of Sports Medicine, Institute of Sports Sciences, Goethe-University Frankfurt, Ginnheimer Landstr. 39, D-60487 Frankfurt am Main, Germany; 2 Department of TCM/Acupuncture, Institute of Complementary Medicine, University of Bern, Freiburgstr. 46, CH-3010 Bern, Switzerland; 3 Sport and Exercise Science Research Centre, School of Applied Sciences, London South Bank University, 103 Borough Rd, SE1 0AA London, UK

**Keywords:** Injection therapy, Return to play, Procaine, Inflammatory pain, Sympathetic nerve, Multimodal treatment

## Abstract

**Background:**

The focus of this case report is on the role of inflammation as a contributor to pain in plantar fasciitis and its cure by the injection of local anesthetics.

**Case presentation:**

This is a case report on a 24-year-old white man, a middle-distance runner, with chronic unilateral plantar fasciitis and perceived heel pain for almost 1.5 years. He was treated with neural therapy (that is, injection of < 1 ml procaine 1% which is a local anesthetic with strong anti-inflammatory properties) of the surgical scar and along the surgical puncture channel. The follow-up period from the time of first presentation until publication was 2.5 years. At admission, pain intensity (visual analog scale) in the affected leg was severe (10 cm, visual analog scale; range 0–10 cm) when walking and moderate (5 cm, visual analog scale) when standing. After the first session of injections he could stand pain-free and pain when walking was markedly reduced (− 90%). After the third session, he reported no pain in the affected leg and could return to sports at his former level (no difference in training load compared to non-injured state). There was no recurrence of inflammatory signs or heel pain despite intense athletics training up to the date of publication.

**Conclusions:**

In prolonged cases of plantar fasciitis, inflammation is an important component in the development of persistent pain. The results of our case describe the effects of three neural therapy sessions that abolished inflammation and associated heel pain. Neural therapy might be an effective and time-efficient approach in the treatment of plantar fasciitis, enabling an early return to sports.

## Background

Plantar heel pain is pain surrounding the calcaneus; it is most commonly felt posteriorly or inferiorly. Plantar heel pain is one of the most common soft tissue disorders of the foot in sports [1]. Plantar fasciitis has been described as a common cause of heel pain, and has been reported to account for 15% of all adult foot complaints requiring professional care. The prevalence rate in athletes was reported to be 21.7% [2]. Plantar fasciitis has been described in runners in particular; it is an overuse injury associated with the accumulation of repetitive force over time [3]. Morphologically, there is a significant association between plantar fasciitis and calcaneal spur formation, that is, an insertion tendinopathy of the plantar aponeurosis [4]. Plantar fasciitis is thought to occur due to excessive cumulative strain at the enthesis of the plantar fascia. There is a general consensus within the literature that mechanical overload and excessive strain produce microscopic tears within the fascia, which subsequently invoke an inflammatory repair process [5]. The normative repair process is thought to be inhibited by continued microtrauma arising from repeated heel strike, resulting in chronic inflammation of the fascia [5]. Inflammation of the attachment of the plantar fascia is also a major cause of pain [6]. Many researchers consider plantar fasciitis a degenerative tissue condition rather than inflammation [7], the occurrence of pain is supposed to be linked to persistence of inflammatory processes. Autoinflammatory disorders are considered to have a strong pain component [8]. It is known from animal models that fascial inflammation sensitizes dorsal horn neurons that are related to the appearance of new receptive fields [9], which leads to enlarged pain sensitivity.

Several options have been proposed in the therapy of plantar fasciitis. The Clinical Practice Guideline Heel Pain Panel of the American College of Foot and Ankle Surgeons published some recommendations in 2010 [10], summarizing the body of evidence at that time. The authors suggested padding and stretching of the foot, therapeutic orthotic insoles, orally administered anti-inflammatory medication, injections of corticosteroids, and stretching of Achilles and plantar fascia as first-line therapies. This judgment was made on the basis of level II and IV studies (Grade B recommendations). If these approaches fail within 6 weeks, second-line options include the use of orthotic devices, night splints, repeated corticosteroid injection, injection of botulinum toxin, a course of physical therapy, and cast immobilization (maximum evidence Grade B [10]). In obese patients, a weight loss program seems to be beneficial. First-line treatments can be continued. Options improving the patients’ state should be continued until the problem is resolved. Third-line approaches include endoscopic plantar fasciotomy or minimally invasive surgery, shockwave therapy, or bipolar radiofrequency [10]. The use of surgical interventions is seen appropriate when conservative methods fail within 6 months [11, 12].

Most causes of plantar fasciitis can be successfully managed non-surgically [12]. A broad range of techniques can be incorporated under the term physical therapies. Besides extracorporeal shockwave therapy (ESWT) and radiofrequency thermal lesioning [13], there is acupuncture [14] and related techniques such as dry needling [15], as well as the injection of substances other than corticosteroids [16], which have been described as beneficial. Corticosteroid injections should be used with caution as multiple injections have been associated with fascia weakness and rupture [17]. A peppering injection of the local anesthetic prilocaine as well as of autologous blood was shown to be as effective as steroids [16]. This diffuse approach to plantar fasciitis and its limited evidence makes it difficult to draw clear concise conclusions upon which to base clinical practice [18].

## Case presentation

### Patient information

**Fig. 1:**
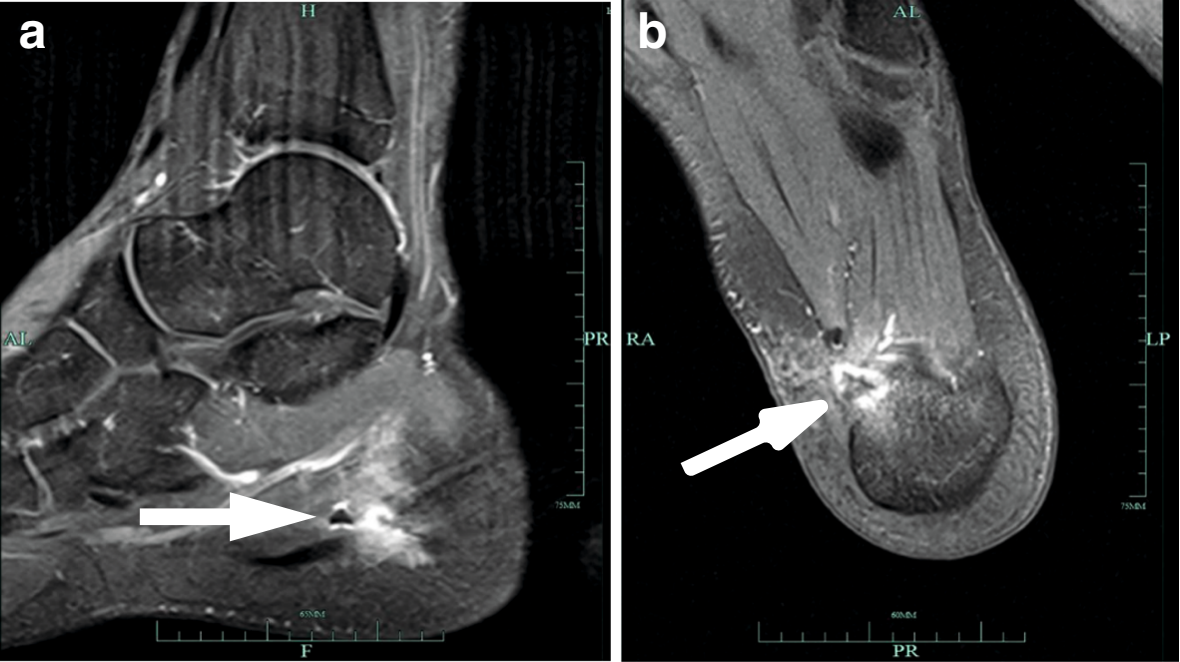
T1-weighted MRI. **a** Sagittal view. **b** Horizontal view. Figures show a minor calcaneal spur, with severe signs of inflammation at the calcaneal tubercle (see *arrows*) surrounded by a large edema, and structural changes of the plantar fascia

**Fig. 2:**
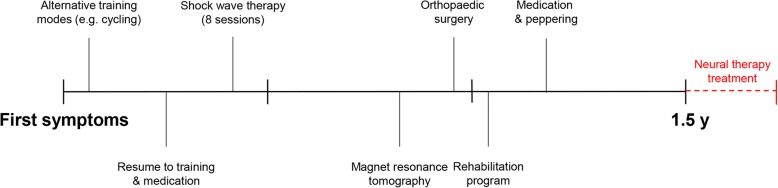
Represents the patient’s timeline before his admission to our out-patient clinic. *y* year

A 24-year-old white man, a middle-distance runner (800 m) competing at national level (seasonal best/personal best of 1 minute 52 seconds), developed severe left heel pain in the pre-season in March 2013. His maximum perceived pain intensity was 10 cm on a visual analog scale (VAS) that ranged from 0 to 10 cm, with 10 cm expressing the worst perceivable pain; the athlete had to interrupt all running activity, and severe pain was perceived even when walking or standing. He continued training with aqua jogging and cycling. He got personalized hand-crafted orthopedic gel peads. Two months later he was attended by an orthopedic surgeon, who additionally prescribed oral intake of nonsteroidal anti-inflammatory drugs (NSAIDs) for 8 weeks. The athlete could continue his training but was not free from pain. When discontinuing medication in July 2013, pain returned immediately, and perceived pain intensity during walking was 10 cm on a VAS (range 0–10 cm). Eight sessions of ESWT were thus added to his treatment plan, and were conducted at a German Olympic center. He did not feel better after the treatment and reported a high level of frustration. An MRI was performed in January 2014 showing a calcaneal spur, signs of inflammation at the calcaneal tubercle, and structural changes of the plantar fascia, surrounded by a large edema (see Fig. 1). In February 2014 he underwent an open plantar fasciotomy. Four weeks later he was allowed to perform the first units of regenerative running. Pain returned after approximately 1 week of training. An X-ray revealed no pathology and he was recommended to continue with soft training sessions. He received a peppering injection that reduced pain for 12 hours, and NSAIDs were re-prescribed. His running performance remained at a remarkably low level in comparison to his non-injured state, despite regular personalized training sessions. He presented himself at our out-patient clinic in July 2014 (for timeline see Fig. 2).

### Diagnostic assessment

An examination identified pain to palpation at the medial calcaneal tubercle and along the medial band of the plantar fascia. Thickening and enlargement of the proximal one-third plantar fascia was noted. Full and pain-free range of motion was noted to his ankle and foot. Standing caused moderate (VAS score, 5 cm) pain; walking caused severe (VAS score, 10 cm) pain. Latent myofascial trigger points could be found in the surrounding muscles: gastrocnemius medialis and lateralis, and tibialis posterior. Apart from these symptoms no abnormalities in his medical or family history which may have been relevant to the medical case were reported and he presented himself in a good mental condition. The diagnosis based on these findings was chronic plantar fasciitis (calcaneal spur syndrome).

### Therapeutic intervention

He was treated with neural therapy (that is, injection of < 1 ml procaine 1%, which is a local anesthetic) of the surgical scar and along the surgical puncture channel. He lay in a supine position on a treatment table. Sessions took approximately 5 minutes. In total, three sessions (at baseline, at week 1, and after 4 weeks) were performed.

### Outcomes

At the first treatment (March 2015), he described a slurping noise, like “if something filled up the pain origin.” Afterwards he could stand pain-free and walking (not running) was subjectively improved. After the third session the pain had been completely eliminated (VAS = 0 cm). He could return to sports at the former level. Since March 2015 no recurrence of the problem could be observed. No adverse events were observed.

## Discussion

Plantar fasciitis and associated heel pain in sports medicine are common conditions. The present case details a typical athlete’s career in which the athlete encountered pain that stubbornly resisted different treatment approaches. Even though there is a predominantly consistent pathophysiological model, many different treatment modalities were recommended to address his needs. Several cases reported over the years show the uniqueness of each individual case, for example: a female collegiate basketball player who returned to sports 12 weeks after ESWT [18]; a female recreational runner with gait training [19]; or a retired male golfer and hockey player, treated via percutaneous ultrasonic fasciotomy, who returned to all activities at 4 weeks [20]. A common factor to all these cases is that the treatment approaches initiated first could reduce the athletes’ symptoms. However, reports on chronic courses are sparse.

We supposed an increased inflammatory stress response inside the athlete’s heel area, which is a major cause of pain chronification. In the present case, we opted to try injections with therapeutic local anesthesia (neural therapy) subcutaneous to the scar and into the depth along the surgical puncture channel. The purpose of this technique is not primarily to achieve local anesthesia. Neural therapy utilizes the regulatory mechanisms and plastic properties of the nervous system. The generation of targeted mechanical stimuli (through the needle) and the selective extinction of other stimuli (through the local anesthetic) affect both the organization of the nervous system and tissue perfusion, thereby disrupting positive feedback actions (vicious circle including a complex interplay of nociceptor activity, sympathetic excitation, circulation disturbance, neurogenic inflammation, and muscle hardening) in the pain cycle [21]. The reduction of perceived pain after the first session is a common observation in neural therapy; it is considered a beneficial diagnostic sign that indicates that the neural therapist should continue with additional sessions [21]. The aim of further sessions is to abolish the above-mentioned pain-contributing mechanism. In fact, repeated application of local anesthetics has been shown to directly reduce neurogenic inflammation [22].

To the best of our knowledge there is only one non-sports-related report on a 37-year-old male duty service member with bilateral heel pain over several years [23] that focused on nervous irritation. Here, the patient received an ultrasound-guided injection with fluid tissue dissection of the nerve from surrounding tissue with 2 mL of lidocaine, resulting in a 90% improvement in the morning heel pain intensity. In our current approach, we did not aim to block the nerve, but to abolish local pain mechanisms. Our attempt aimed to hit two structures:

1. The scarring of the skin resulting from surgery. It was proposed that scars transmit signals by, for example, deforming the skin’s collagen fibers, and are a source for the ectopic generation of action potentials [24]. Subcutaneous infiltration with local anesthetics has been reported to have a favorable effect on the painfulness by reducing the peripheral sensitization [24].
2. The wound cavity. This is the focus of inflammation according to imaging and surgical intervention.

The link between pain and inflammation has been described as “inflammatory reflex of the autonomic nervous system” [25]. Interrupting this reflex with the injection of the anti-inflammatory local anesthetic procaine should help to restore the physiologic conditions of the tissue [22]. In this context it is important to note that our patient underwent various treatments before neural therapy was applied, potentially limiting the validity of the current findings. However, as former rehabilitation programs were completed approximately 13 months before our treatment and the injections were conducted within a short time period of only 1 month, we are confident that the applied neural therapy was the primary driver for the disappearance of symptoms (that is, heel pain and inflammatory signs) in the current case.

## Conclusions

In prolonged cases of plantar fasciitis, the inflammatory component of the disease may play a major role in the development of persistent pain and be a target goal for interventions. Locally applied therapies, which aim to reduce the inflammatory state, seem superior to a systemic drug because they will directly act on the pathophysiologically altered tissue. In this case report, an injection with the anti-inflammatory-acting local anesthetic procaine completely resolved the irritation around the calcaneus, sustainably enabling our patient to return to sports at a former level, within a potentially very short time period.

### Patient’s perspective

Before heel pain emerged for the first time during the track and field camp in March 2013 I performed 800 and 1500 m at a national level (personal best, 1 minute 52 seconds). After a few weeks of training, my heel pain became worse and remained even during normal walking and everyday activities, requiring alternative training modes (that is, cycling and aqua jogging). After a longer period of regular running training in combination with medication, the pain returned and became worse. Following this, shock wave therapy and physical therapy were performed at my respective Olympic Training Center, showing no effect. A MRI and surgery was performed in February 2014, which was followed by an immediate rehabilitation program (first running sessions approximately 4 weeks after the surgery). The first training sessions were very promising but my heel pain returned after 1 week of regenerative training. Again, I perceived pain during both running and normal walking, preventing any running ambitions for the new season (even despite continuous medication). After a few sessions of neural therapy, the inflammation-like pain was eliminated and I could resume running training at a high level. Since the neural therapy was completed, at no time have I had problems again within the affected heel even after high intense training sessions.
