# Correction to: Neural therapy of an athlete’s chronic plantar fasciitis: a case report and review of the literature

**DOI:** 10.1186/s13256-018-1852-3

**Published:** 2018-10-01

**Authors:** J Fleckenstein, M König, W Banzer

**Affiliations:** 1 Department of Sports Medicine, Institute of Sports Sciences, Goethe-University Frankfurt, Ginnheimer Landstr. 39, 60487 Frankfurt am Main, Germany; 2 Department of TCM/Acupuncture, Institute of Complementary Medicine, University of Bern, Freiburgstr. 46, CH-3010 Bern, Switzerland; 3 Sport and Exercise Science Research Centre, School of Applied Sciences, London South Bank University, 103 Borough Rd, London, SE1 0AA UK

## Correction

In the publication of this article [1], there are reference errors in four positions the respective references are missing since reference Fischer [26] was omitted. In addition for reference Egli *et al.* [21] the in text citation only appeared at the end of the paragraph, but not following important statements. This has now been included in this correction.

In the Discussion section it currently reads:

“The purpose of this technique is not primarily to achieve local anesthesia. Neural therapy utilizes the regulatory mechanisms and plastic properties of the nervous system. The generation of targeted mechanical stimuli (through the needle) and the selective extinction of other stimuli (through the local anesthetic) affect both the organization of the nervous system and tissue perfusion, thereby disrupting positive feedback actions (vicious circle including a complex interplay of nociceptor activity, sympathetic excitation, circulation disturbance, neurogenic inflammation, and muscle hardening) in the pain cycle [21].”

In the Discussion section it should instead read:

“The purpose of this technique is not primarily to achieve local anesthesia [21, 26]. Neural therapy utilizes the regulatory mechanisms and plastic properties of the nervous system [21, 26]. The generation of targeted mechanical stimuli (through the needle) and the selective extinction of other stimuli (through the local anesthetic) affect both the organization of the nervous system and tissue perfusion [21, 26], thereby disrupting positive feedback actions (vicious circle including a complex interplay of nociceptor activity, sympathetic excitation, circulation disturbance, neurogenic inflammation, and muscle hardening) in the pain cycle [21, 26].”
